# Lycopene Exerts Neuroprotective Effects After Hypoxic–Ischemic Brain Injury in Neonatal Rats via the Nuclear Factor Erythroid-2 Related Factor 2/Nuclear Factor-κ-Gene Binding Pathway

**DOI:** 10.3389/fphar.2020.585898

**Published:** 2020-11-24

**Authors:** Changchang Fu, Yihui Zheng, Jinjin Zhu, Binwen Chen, Wei Lin, Kun Lin, Jianghu Zhu, Shangqin Chen, Peijun Li, Xiaoqin Fu, Zhenlang Lin

**Affiliations:** ^1^ Department of Neonatology, The Second Affiliated Hospital and Yuying Children’s Hospital of Wenzhou Medical University, Wenzhou, China; ^2^ School of Second Clinical Medical, Wenzhou Medical University, Wenzhou, China; ^3^ University of Illinois at Chicago, College of Pharmacy, Chicago, IL, United States; ^4^ The Second Affiliated Hospital and Yuying Children’s Hospital of Wenzhou Medical University, Wenzhou, China

**Keywords:** poptosis, neuroprotection, lycopene, hypoxic-ischemic brain injury, Nrf2/NF-κB

## Abstract

Neonatal hypoxic-ischemic encephalopathy (HIE) is a brain injury caused by perinatal asphyxia and is the main cause of neonatal death and chronic neurological diseases. Protection of neuron after hypoxic-ischemic (HI) brain injury is considered as a potential therapeutic target of HI brain injury. To date, there are no effective medicines for neonatal HI brain injury. Lycopene (Lyc), a member of the carotenoids family, has been reported to have anti-oxidative and anti-inflammatory effects. However, its effects and potential mechanisms in HI brain injury have not yet to be systematically evaluated. In this study, we investigated whether Lyc could ameliorate HI brain injury and explored the associated mechanism both *in vivo* and *in vitro* experiments. *In vivo* study, Lyc significantly reduced infarct volume and ameliorated cerebral edema, decreased inflammatory response, promoted the recovery of tissue structure, and improved prognosis following HI brain injury. *In vitro* study, results showed that Lyc reduced expression of apoptosis mediators in oxygen-glucose deprivation (OGD)-induced primary cortical neurons. Mechanistically, we found that Lyc-induced Nrf2/NF-κB pathway could partially reversed by Brusatol (an Nrf2 inhibitor), indicated that the Nrf2/NF-κB pathway was involved in the therapy of Lyc. In summary, our findings indicate that Lyc can attenuated HI brain injury *in vivo* and OGD-induced apoptosis of primary cortical neurons *in vitro* through the Nrf2/NF-κB signaling pathway.

## Introduction

Hypoxic-ischemic (HI) brain injury is associated with high morbidity and mortality rate in neonates. Therefore, HI brain injury is a problem that needs to be urgently prevented and treated (Hochwald et al., 2014). It is reported that incidence of neonatal HI encephalopathy (HIE) is 1–8 of every 1,000 live births in developed countries (Yıldız et al., 2017). The rate is higher in developing countries (Douglas-Escobar and Weiss, 2015). Mild hypothermia is the most commonly used clinical treatment and has been demonstrated to decrease the fatality rate of HI brain injury and sequelae of the nervous system (Lin et al., 2006).

However, mild hypothermia is also accompanied by an economic burden to patients and society (du Plessis and Volpe, 2002). Therefore, effective and safe therapy for neonatal HI brain injury is required. The mechanism of neonatal HI brain injury is not completely revealed. In previous studies, inflammation has been demonstrated to play a pivotal role in HI brain injury (Perlman Similarly, 2006; Hagberg et al., 2015). Some reports suggested that apoptotic and inflammatory processes play a pivotal role in the pathophysiology of ischemic brain injury (Lindsberg and Grau, 2003). Riljak et al. (2016) have found that inflammatory responses occur in cases of perinatal HIE (Price et al., 2004). Furthermore, many experiments have uncovered that the postischemic brain inflammatory responses were principally characterized by rapid activation of resident cells followed by the infiltration of circulating inflammatory cells (Price et al., 2004). In addition, apoptosis has been demonstrated to be essential across various diseases of the central nervous system (Northington et al., 2011). Several studies have confirmed that apoptosis leads to delayed death of developing brain cells, causing massive cell loss and neurodegeneration (Rocha-Ferreira and Hristova, 2016). Thus, lessening apoptosis can serve as a useful therapeutic target following hypoxic-ischemic.

In the development of neonatal HI brain injury, the NF-κB signaling pathway regulation of inflammatory mediators has been demonstrated as a pivotal pathway (Fang et al., 2018). Prior studies have identified that the NF-κB pathway could be inhibited by Nrf2 (Ahmed et al., 2017). When stimulated with Oxidative Stress, Nrf2 separates from Keap1 and translocates into the nucleus. Then, Nrf2 will up-regulate the expression of HO-1. Finally, HO-1 suppresses inflammation response by inactivating P65, a unit of NF-κB (Jiang et al., 2015; Zhang et al., 2006). Under basal conditions, P65 is inactive and combines with the inhibitory protein IκBα when localized in the cytoplasm (Li et al., 2016). After oxygen-glucose deprivation (OGD) stimulation, P65 becomes activated and translocates into the nucleus (Jiang et al., 2015). Furthermore, phosphorylation of P65 allows it to be translocated from the cytoplasm into the nucleus where it upregulates expression of inflammatory factors such as tumor necrosis factor α (TNF-α), Interleukin- 6 (IL-6) and inducible nitric oxide synthase (iNOS) (Liang et al., 2017). These inflammatory factors are considered to be the chief contributors to ischemic brain injury (Williams et al., 2006). Thus, strategies targeting multiple molecular pathways are extensively useful for reducing HI-induced neuronal damage.

Lycopene (Lyc) is a member of the carotenoids family (Olson and Krinsky, 1995; Condori et al., 2020), mainly exists in fruits and vegetables including tomatoes, watermelon, and red pomelo (Mangels et al., 1993). Lyc is especially high in the red variety of tomatoes, and the structure remains in food processing. In recent years, studies on the therapeutic effects of carotenoids in human diseases have aroused much attention, especially in Lyc, owing to its efficacy and safety. Studies have shown that carotenoids have certain effects on human health and diseases, including cardiovascular protection and inhibiting the proliferation of malignant tumors like prostate cancer and breast, etc (Frei, 1995; Giovannucci et al., 1995). It had also been reported that Lyc could play an anti-inflammatory role in mouse airway inflammation and has an anti-inflammatory effect on intestinal inflammation in the rat (Lee et al., 2008). Some studies demonstrated that the mechanism of Lyc protects Leydig cell from damage may be that the Nrf2 pathway was regulated to exert anti-inflammation and antioxidant effect (Zhao Y. et al., 2020). In addition, some studies have pointed out that Lyc could down-regulate TNF- α and IL-6, thus inhibiting the phosphorylation of P65, and enhancing expression of Nrf2 (Zhao Q. et al., 2020). Moreover, previous studies suggest that Lyc has anti-inflammatory and immunomodulatory abilities in H_2_O_2_-induced SH-SY5Y cells (Zhao et al., 2017).

However, there have only been a few studies on Lyc in HIE. Therefore, we conducted a study to investigate the neuroprotective effect of Lyc in neonatal brain damage induced by HI injury, and determined whether the Nrf2/NF-κB signaling pathway is involved in this process.

## Materials and Methods

### Reagents

Lyc (purity ≥98%) was purchased from Solarbio (Wuhan, China). Dimethylsulfoxide (DMSO), and poly-D-lysine were obtained from Sigma Chemical Co. (St. Louis, MO, United States). Fetal bovine serum, B27, neurobasal medium, 0.5 mM L-glutamine and Dulbecco’s modified Eagle medium obtained from Gibco (Grand Island, NY, United States). Primary antibodies: TNF-a (ab66579; Abcam, Cambridge, United Kingdom), Bcl-2 (ab196495; Abcam, Cambridge, United Kingdom), Bax (ab32503; Abcam, Cambridge, United Kingdom), Cleaved caspase-3 (ab49822; Abcam, Cambridge, United Kingdom), GAPDH (10494-1-AP; Proteintech, Wuhan, China), Lamin B (12987-1-AP; Proteintech, Wuhan, China), IkBa (#9242; Cell Signaling Technology, MA, United States), P65 (#8242; Cell Signaling Technology, MA, United States), Nrf2 (#12721; Cell Signaling Technology, MA, United States), HO-1 (#43966; Cell Signaling Technology, MA, United States). The secondary antibodies of Goat Anti-Rabbit IgG and Alexa Fluor^®^488 labeled were obtained from Bioworld (OH, United States). Cell-Counting Kit-8 (CCK-8) was purchased from Dojindo (Kumano, Japan). The nuclear stain 4′,6-diamidino-2-phenylindole (DAPI) was purchased from Beyotime (Shanghai, China). Bovine serum albumin (BSA) was procured from Beyotime Biotechnology (ShangHai, China).

### Neonatal Hypoxic-Ischemic Brain Injury Model and Drug Administration

Sprague Dawley (SD) rats (200–250 g) were obtained from the Animal Center of the Chinese Academy of Sciences Shanghai, China. The protocols of animal use and care and the experimental procedures were performed in accordance with the Animal Care and Use Committee of Wenzhou Medical University. Adult SD rats were allowed to freely mate, and postnatal day 7 (P 7) male pups are used for experiments. According to prior reports, the modified Rice-Vannucci model was used (Taniguchi and Andreasson, 2008). Isoflurane was used to completely anesthetize and maintain the P 7 pups. Then, the left common carotid artery of P 7 pup was separated, ligated and cut within 5 min. The pups then recovered in the dam for 2 h post-surgery. After getting enough rest, the pups were placed into a humid mixed gas chamber composed of 92% N_2_ and 8% O_2_ and ventilated at a flow rate of 3 L/min for 2 h. Place the above chamber in a constant temperature water bath at 37.5°C. The pups in the sham surgery group were not ligated with common carotid arteries and were not hypoxic. After the end of hypoxic, all pups were returned to their cages for subsequent experimental procedures. In the following days, the rats were given daily administration of the drugs. The Lyc treatment group received intragastric administration of different concentrations of Lyc (5, 10, or 20 mg/kg) immediately after hypoxic at 24 h intervals until the pups were euthanized in order to determine the most effective concentration. The Brusatol + Lyc group were administered Lyc (10 mg/kg) and Brusatol (0.4 mg/kg).

### Infarct Volume Measurement

We used the 2,3,5-triphenyl tetrazolium chloride (TTC) staining to measure the infarct volume, as the previous study described (Wang et al., 2020). The rat brain tissues were collected 24 h after HI brain injury, and stored at −20°C for 12 min and sectioned into 2-mm-thick coronal slices. Then, the samples were incubated with a 1% TTC (Sigma, Unites States) solution at 37°C in the dark for 30 min and then changed the solution to 4% PFA for 24 h. ImageJ software was used to measure the cerebral infarct volume.

### Morris Water Maze Test

We used the Morris Water Maze (MWM) test to evaluate the learning and memorizing ability of experimental animals. The MWM test is an experiment that compels animals to swim in search of a platform hidden underwater. We conducted the MWM test 21 days after the HI brain injury (Zhou et al., 2020). In short, rats were swimming in a black circular pool with a diameter of 140 cm and a height of 50 cm. The depth of water in the pool was 1 cm higher than the movable platform. We dye the water black with non-toxic black ink. Next, we divided the pool into four equal quadrants. The pool was located in a room unaffected by noise and light. The MWM test lasted 6 days. The experiment was conducted with the SLY-WMS Morris water maze experiment system.

### Quantitative Real-Time-PCR

After HI injury, the total RNA of brain tissues was obtained using the TRIzol Reagent (Invitrogen). Quantitative real-time PCR (qRT-PCR) was conducted using CFX96Real-TimePCR System (Bio-Rad Laboratories, California, Unites States). The experiment was carried out under the following conditions: 10 min 95°C, followed by 40 cycles of 15 s 95°C and 1 min 60°C. The reaction was carried out in a total of 10 μl, containing 5 µl of 2 × SYBR Master Mix, 0.25 µl of each primer and 4.5 µl of diluted cDNA. Cycle threshold (Ct) values were collected and normalized to the GAPDH levels. Relative mRNA levels of each target gene were calculated using the 2^−ΔΔ^ Ct method. TNF-a, IL-18, IL-6 and iNOS primers were designed using the NCBI Primer-Blast Tool. The forward and reverse primer sequences are shown in Table 1.

**TABLE 1:** The primer sequences used for real-time PCR.

Gene	Forward primers	Reverse primers
TNF-α	TACTCCCAGGTTCTCTTCAAGG	GGAGGCTGACTTTCTCCTGGTA
INOS	AGGCCACCTCGGATATCTCT	GCTTGTCTCTGGGTCCTCTG
IL-18	AAACCCGCCTGTGTTCGA	TCAGTCTGGTCTGGGATTCGT
IL-6	-GAGTTGTGCAATGGCAATTC	ACTCCAGAAGACCAGAGCAG

### Histopathological Analysis

The samples were collected 7 days after the HI injury. We anesthetized the rats 7 days after HI injury, and conducted the cardiac perfusion with 20 ml sterile saline and then perfused with 4% PFA. The samples were then immersed in 4% PFA and store at 4°C for 24 h. The samples were then embedded in paraffin, and cut into coronal sections for subsequent histological analysis, which intuitively displays the hemispheric integrity of the functional neurons between the cerebral cortex and the hippocampus. The above brain slices were dewaxed, hydrated, and stained using H&E or Nissl solution (Solarbio, Beijing, China). Finally, we used an optical microscope to measure the results of the histological staining and analyzed the results using ImageJ software.

### Isolation and Culture of Primary Cortical Neurons

Adult rats were allowed to freely mate in order to produce offspring for subsequent studies. In brief, the primary cortical neurons were prepared from embryonal brains (E16–18 d) of SD rats (Ye et al., 2019). First, we separated the cerebral cortices and washed with PBS three times. Second, the samples were digested using 0.25% trypsin-EDTA solution for 10 min at 37°C. Third, the suspend samples were filtered using a cell strainer. Then, the culture plates were coated with poly-D-lysine overnight, onto which cells were seeded at 1 × 10^6^ cells/mL in 24-well and 96-well culture plates. The cells were then cultured in an environment containing 5% CO_2_ at 37°C. Next, cells were cultured in DMEM medium with 10% FBS under an atmosphere containing 5% CO2 at 37°C for 6 h. Finally, the cell medium was changed to neurobasal medium containing 2% B27, 0.5 mM L-glutamine, 50 U/ml penicillin and 50 μg/ml streptomycin.

### Oxygen-Glucose Deprivation Model

The primary cortical neurons were initially maintained under an anoxic (95% N_2_ and 5% CO_2_) condition to induce OGD insult in serum/glucose-free DMEM medium at 37°C for 3 h. After inducing OGD insult, the culture medium was replaced by neurobasal medium. Next, the cells were divided into separate groups including the OGD group without Lyc, the OGD + Lyc group (addition of 2.5,5,10 μM Lyc), and the OGD + Lyc + Brusatol (an Nrf2 inhibitor) group (addition of 10 μM Lyc and 100 nM Brusatol) (Fan et al., 2020). The plates were then transferred to a normoxic incubator with 95% air and 5% CO_2_ for 24 h.

### Cell Viability

The cytotoxicity of Lyc on primary cortical neurons was measured using the CCK-8 kit (CCK-8; Dojindo Co., Kumamoto, Japan) according to the protocol of the manufacturer. First, the primary cortical neurons were cultured in 96-well plates (8,000 cells/well). Then, the cells incubated with a concentration gradient (0, 2.5, 5, 10, 20, 40 and 80 μM) of Lyc for 24 h. Finally, we added 10 μl CCK-8 solution to each well and incubated 96-well plates at 37°C for 3 h. The absorbance of the wells was then detected at a wavelength of 450 nm using a microplate reader (Leica Microsystem, Germany).

### Immunofluorescence Staining

For cleaved caspase-3 staining, the plates were incubated with the OGD injury for 3 h. The cells rinsed with PBS and treated with 4% paraformaldehyde (PFA) fixation for 15 min, then the wells were washed with PBS three times again. Then, we treated with 0.1% Triton X-100 diluted in PBS for 15 min at indoor temperature. Next, cortical neurons were blocked with 10% goat serum and incubation with primary antibodies against Cleaved caspase-3 (1:300) for the whole night at the temperature of 4°C. The next day, cells were exposed to Alexa Fluor^®^ 488-labeled conjugated secondary antibodies (1:400) for 1.5 h. Finally, the cells were exposed to DAPI for 1 min. Ultimately, cell samples were visualized on the Olympus fluorescence microscope (Tokyo, Japan). The fluorescence intensity was determined using ImageJ software.

### Western Blot

Protein expression in the brain tissues and neurons were measured by Western blotting. The proteins were separated by using RIPA lysis buffer (1 mM PMSF), sonicated on ice for 10 min and then centrifuged at 12,000 rpm at 4°C for 15 min. Then, the protein concentration was evaluated via the BCA protein assay kit (Beyotime). The protein (40 mg) was separated using sodium dodecyl sulfate (SDS)-polyacrylamide gel electrophoresis, followed by transferring to a polyvinylidene difluoride (PVDF) membrane (Millipore). After blocking with 5% nonfat milk for 3 h, the acquired membranes were then incubated with the primary antibodies: Bcl-2 (1:2,000), TNF-a (1:2,000), p65 (1:2,000), Bax (1:2,000), Cleaved caspase-3 (1:2,000), GAPDH (1:2,000), IkBα (1:2,000), IL-18 (1:2,000), Lamin B (1:2,000), Nrf2 (1:2,000) and HO-1 (1:2,000). The following step included incubation with the secondary antibodies at room temperature for 2.5 h. The blots were visualized through the Imaging System (Bio-Rad) followed by washing with TBST 3 times.

### Statistical Analysis

All experiments were performed from at least three independent experiments. Data are presented as mean ± SEM. Statistical analyses were performed using GraphPad Prism version 7.0 software (GraphPad Software, San Diego, CA, Unites States). When analyzing more than two groups, inter-group comparisons were performed using one-way ANOVA followed by the Tukey test. The student’s t-test was used for comparison of two groups. Probability values of *p* < 0.05 were considered statistically significant.

## Result

### Lycopene Attenuated Hypoxic-Ischemic Brain Injury in Neonatal Rats

**FIGURE 1:**
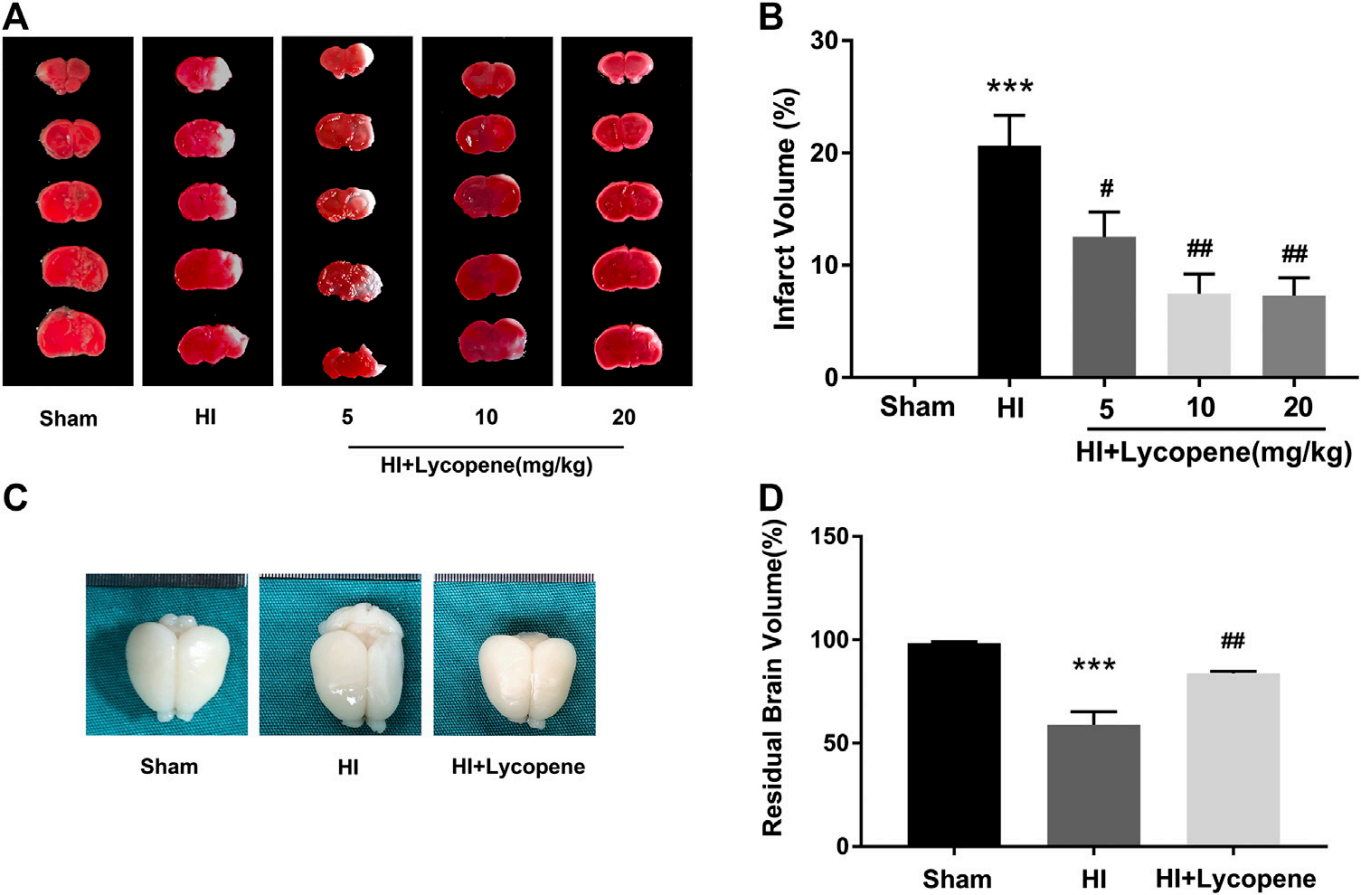
Lycopene treatment attenuated hypoxic-ischemic (HI) brain injury in neonatal rats. **(A)** Representative images of 2,3,5-triphenyl tetrazolium chloride (TTC) staining of coronary brain one day after HI injury. n = 5. **(B)** Analysis of infarct volume based on TTC staining. ***p < 0.001 vs. the sham group, ^**#**^ *p* < 0.05 and ^**##**^ *p* < 0.01 vs. the HI group. All data are presented as mean ± SEM, n = 5. **(C)** Representative images of the brain from each group 7 days after HI injury. n = 6. Scale bar = 1 mm. **(D)** The ratio of the injured hemisphere to contralateral hemisphere is defined as residual brain volume. ***p < 0.001 vs. the sham group. ^**##**^ *p* < 0.01 vs. the HI group. All data are presented as mean ± SEM, n = 6.

In this study, we used a range of Lyc concentrations (5, 10, 20 mg/kg) via intragastric injection to study its role in the process of HI brain injury and determine the most effective drug dose. As shown in Figures 1A,B, results and quantitative analysis of TTC staining showed that all three doses of Lyc could effectively reduce the volume of cerebral infarction. The effect of 10 mg/kg Lyc was similar to the 20 mg/kg dose, therefore, the concentration of 10 mg/kg was selected for subsequent experiments. In addition, we observed the brain anatomy 7 days after the HI injury. As shown in Figures 1C,D, we identified that the injured hemisphere had severe brain atrophy and even liquefaction. The extent of liquefaction and atrophy were alleviated in the Lyc treatment group compared with the HI-induced group. Compared to the sham group, brain atrophy in the HI group was significantly higher. Therefore, the Lyc treatment group can significantly improve brain atrophy.

### Lycopene Protected Against Hypoxic-Ischemic Brain Injury via Down-Regulation of Expression of Inflammatory and Apoptosis Factors

**FIGURE 2:**
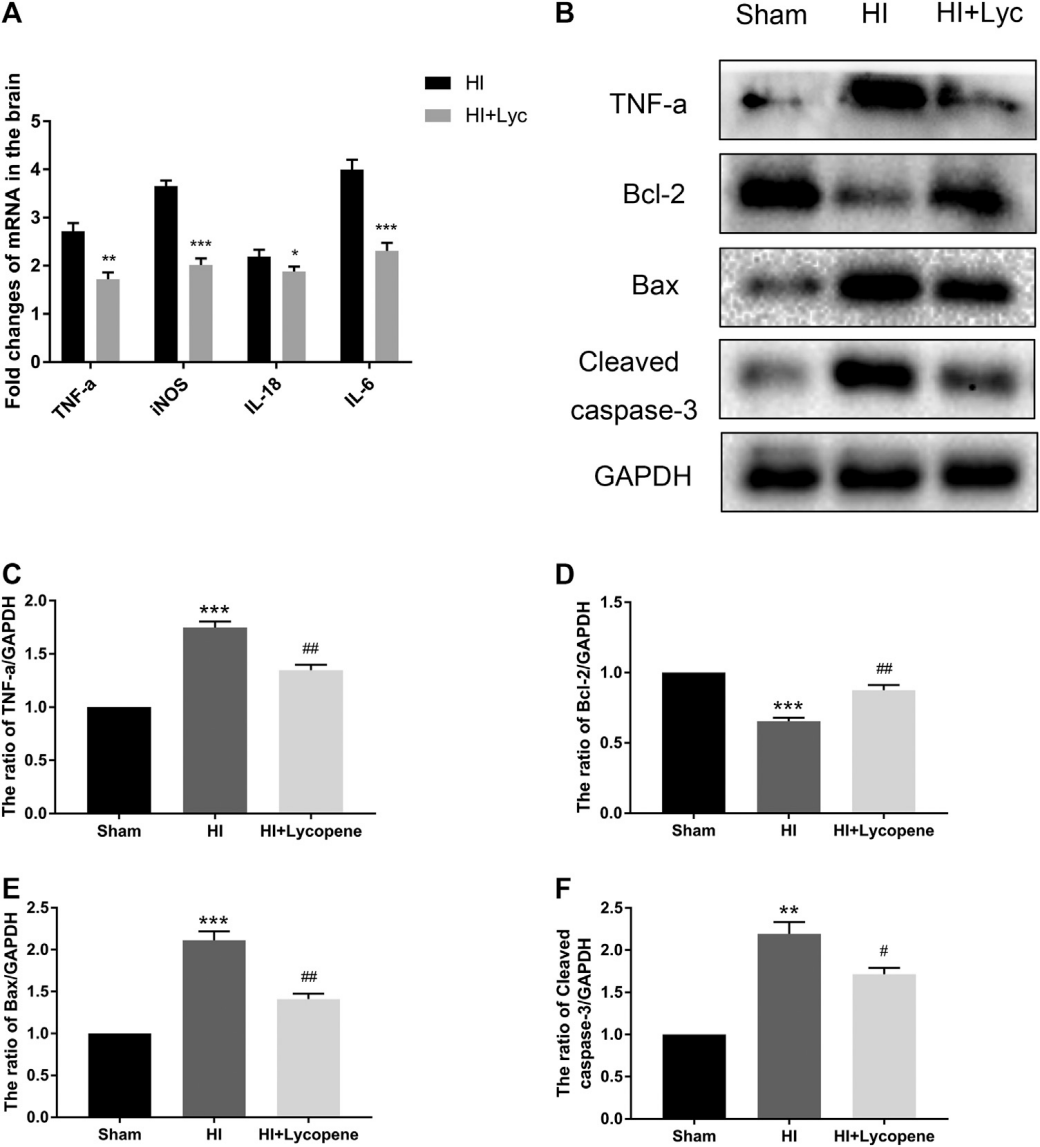
Treatment with Lycopene (Lyc) down-regulated the inflammatory response and apoptosis factors expression after hypoxic-ischemic (HI) brain injury in neonatal rats. **(A)** The mRNA expression of tumor necrosis factor α (TNF-α), interleukin (IL)-18, IL-6 and inducible nitric oxide synthase in brain tissues 24 h after HI injury normalized to those of GAPDH for each sample. **(B)** The protein levels of TNF-α, Bcl-2, Bax and Cleaved caspase-3 were evaluated by Western blot 24 h after HI injury. **(C–F)** Quantitative analysis of the protein levels of TNF-α, Bcl-2, Bax and Cleaved caspase-3 based on Western blot results. **p < 0.01 and ***p < 0.001 vs. the sham group; ^**#**^ *p* < 0.05 and ^**##**^ *p* < 0.01 vs. the HI group. All data are presented as mean ± SEM, n = 3.

Next, we investigated the anti-inflammatory effect of Lyc on HI brain injury through Western blot analysis and RT-PCR. The mRNA expression of TNF-a, IL-18, IL-6 and iNOS in the Lyc group were significantly down-regulated in comparison to the HI group (Figure 2A). The protein expression of TNF-a was consistent with the mRNA levels of TNF-a. Furthermore, we found that Bax and cleaved caspase-3 levels were increased in the HI group compared to the sham group, while Lyc suppressed expression of apoptosis mediators and increased expression of Bcl-2 (Figures 2B–F).

### Lycopene Exerts Neuroprotection in Hypoxic-Ischemic Brain Injury via the Nrf2/NF-κB Signaling Pathway

**FIGURE 3:**
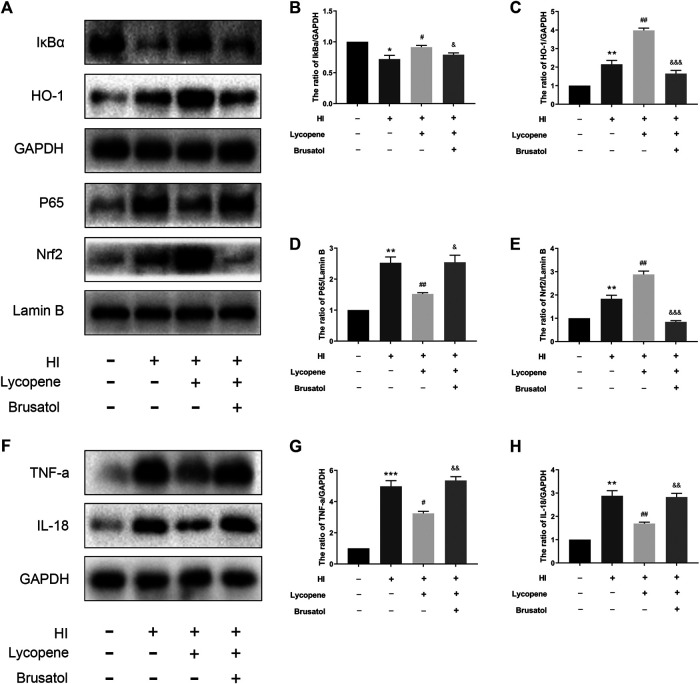
Treatment with Lycopene (Lyc) exerted neuroprotection via the Nrf2/NF-κB signaling pathway after hypoxic-ischemic brain injury in neonatal rats. **(A)**The protein levels of IκBα, HO-1, P65, Nrf2, tumor necrosis factor α (TNF-α) and interleukin (IL)-18 were detected by Western blot 24 h after the HI injury. **(B–H)** Quantitative analysis of IκBα, HO-1, P65, Nrf2, TNF-α and IL-18. *p < 0.05, **p < 0.01 and ***p < 0.001 vs. the sham group; ^#^ *p* < 0.05 and ^##^ *p* < 0.01 vs. the HI group; ^&^ *p* < 0.05, ^&&^ *p* < 0.01 and ^&&&^ *p* < 0.001 vs. the HI + Lyc group. All data are presented as mean ± SEM, n = 3.

Previous studies have revealed that Lyc can reduce the expression of inflammatory mediators by activating the Nrf2/NF-κB signaling pathway. Hence, we used Western Blot to evaluate the effect of Lyc on the Nrf2/NF-κB signaling pathway. Results indicated that levels of P65 increased in the HI group, while levels of HO-1 and Nrf2 slowly increased compared to the sham group (Figures 3A–E). However, the levels of HO-1 and Nrf2 were significantly up-regulated in the Lyc treatment group. Moreover, Lyc treatment suppressed the expression of P65. However, Brusatol (an Nrf2 inhibitor) was able to significantly reduce expression of HO-1 and Nrf2, and increase expression of P65 in the nucleus. Furthermore, TNF-a and IL-18 were down-regulated in the Lyc treatment group compared with the HI group, whereas the protective effect of Lyc was partially reversed by Brusatol (Figures 3F–H). These data indicate that Lyc can inhibit the inflammation in HI brain injury via the Nrf2/NF-κB signaling pathway.

### Lycopene Preserves Brain Tissue Structure After Hypoxic-Ischemic Brain Injury

**FIGURE 4:**
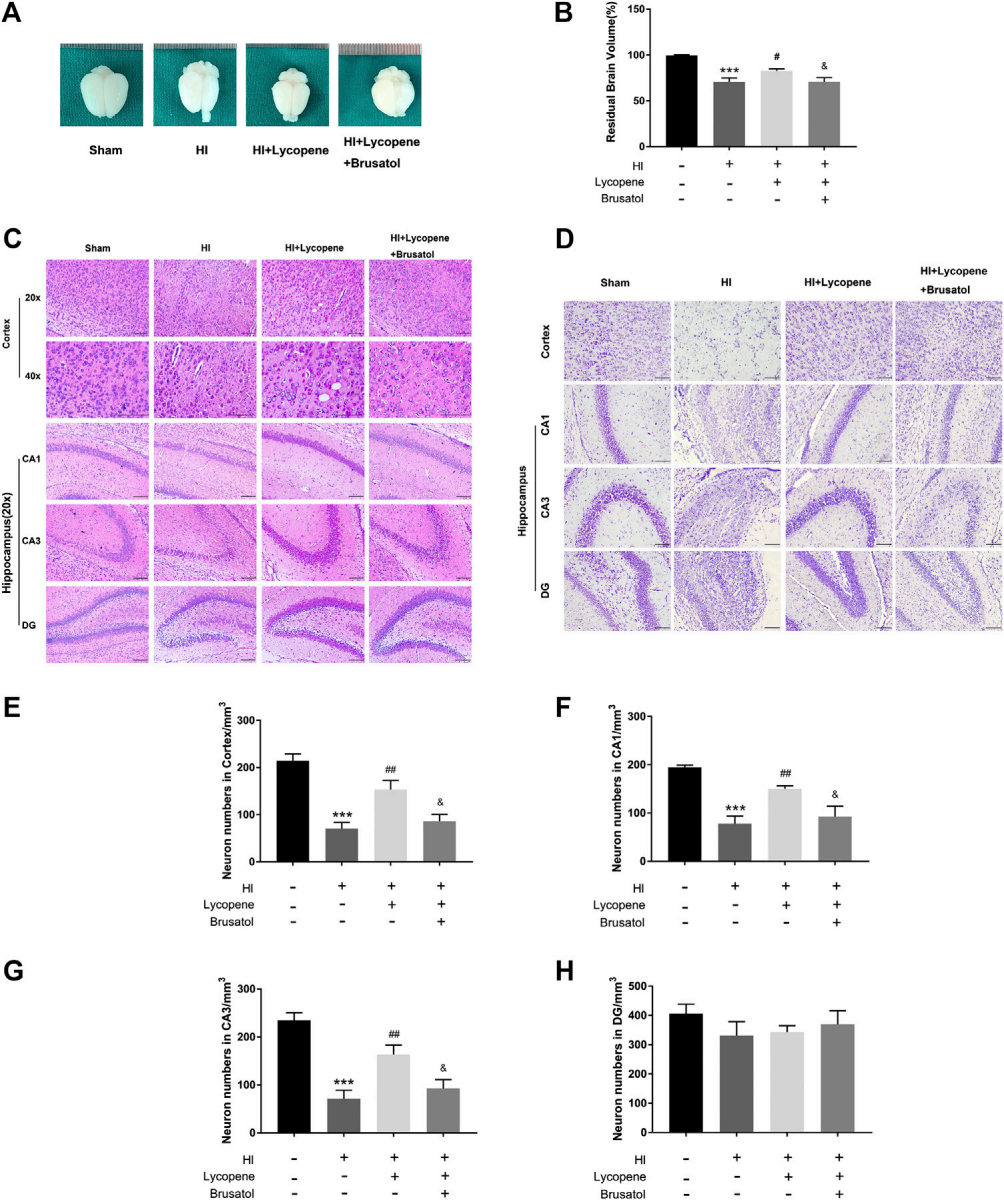
Brusatol reversed the neuro-protective effect of Lycopene (Lyc) after hypoxic-ischemic (HI) brain injury in neonatal rats. **(A)** Representative images of the brain from each group 7 days after HI injury. n = 6. Scale bar = 1 mm. **(B)** The ratio of the injured hemisphere to contralateral hemisphere is defined as residual brain volume. ***p < 0.001 vs. the sham group. ^#^ *p* < 0.05 vs. the HI group. ^&^ *p* < 0.05 vs. the HI + Lyc group. All data are presented as mean ± SEM, n = 6. **(C)** Representative images of H&E staining in the cortex, hippocampus CA1 region, hippocampus CA3 region and DG region 1 week after HI injury. Scale bars = 100 μm. **(D)** Representative images and quantification data **(E–H)** of Nissl staining in the cortex, hippocampus CA1, CA3, DG region 7 days after hypoxic-ischemic injury. Scale bar = 100 μm ***p < 0.001 vs. the sham group; ^##^ *p* < 0.01 vs. the HI group; ^&^ *p* < 0.05 vs. the HI + Lyc group. All data are presented as mean ± SEM, n = 5.

We observed the anatomical structure 7 days after the HI injury. Resemble to the HI group, we found a great extent of liquefaction and atrophy in the HI + Lyc + Brusatol group, which indicated that the neuroprotective effect of Lyc could be inhibited in comparison to the HI + Lyc group (Figures 4A,B).

Furthermore, we conducted H&E staining and Nissl staining to observe neuron-protective effects of Lyc. Histological staining of these brain slices was performed and the HI damage was evaluated by observing the number of cells in different areas. In the sham surgery group, neurons in the cortex, Hippocampus CA1 region, CA3 region, and dentate gyrus (DG) region were oval or round in shape with intact and clear nuclei, and were neatly arranged. Moreover, we found that the Nissl bodies were large and numerous around the nuclei in the sham group. However, in the HI group, neurons suffered from fatal nuclear disorders, and disordered neuronal arrangement, and even neurons were not observed. As for Nissl bodies, they were hardly observed in the brain regions after HI injury. Post-Lyc treatment, the extent of degeneration and necrosis of neuron was markedly decreased, and the numbers of Nissl bodies and neurons increased. Conversely, the increase in neuronal density and morphological recovery after Lyc treatment was partially suppressed by Brusatol. (Figures 4C–H).

### Lycopene Improved the Bodyweight and Learning and Memory Functions of Rats Following Hypoxic-Ischemic Brain Injury

**FIGURE 5:**
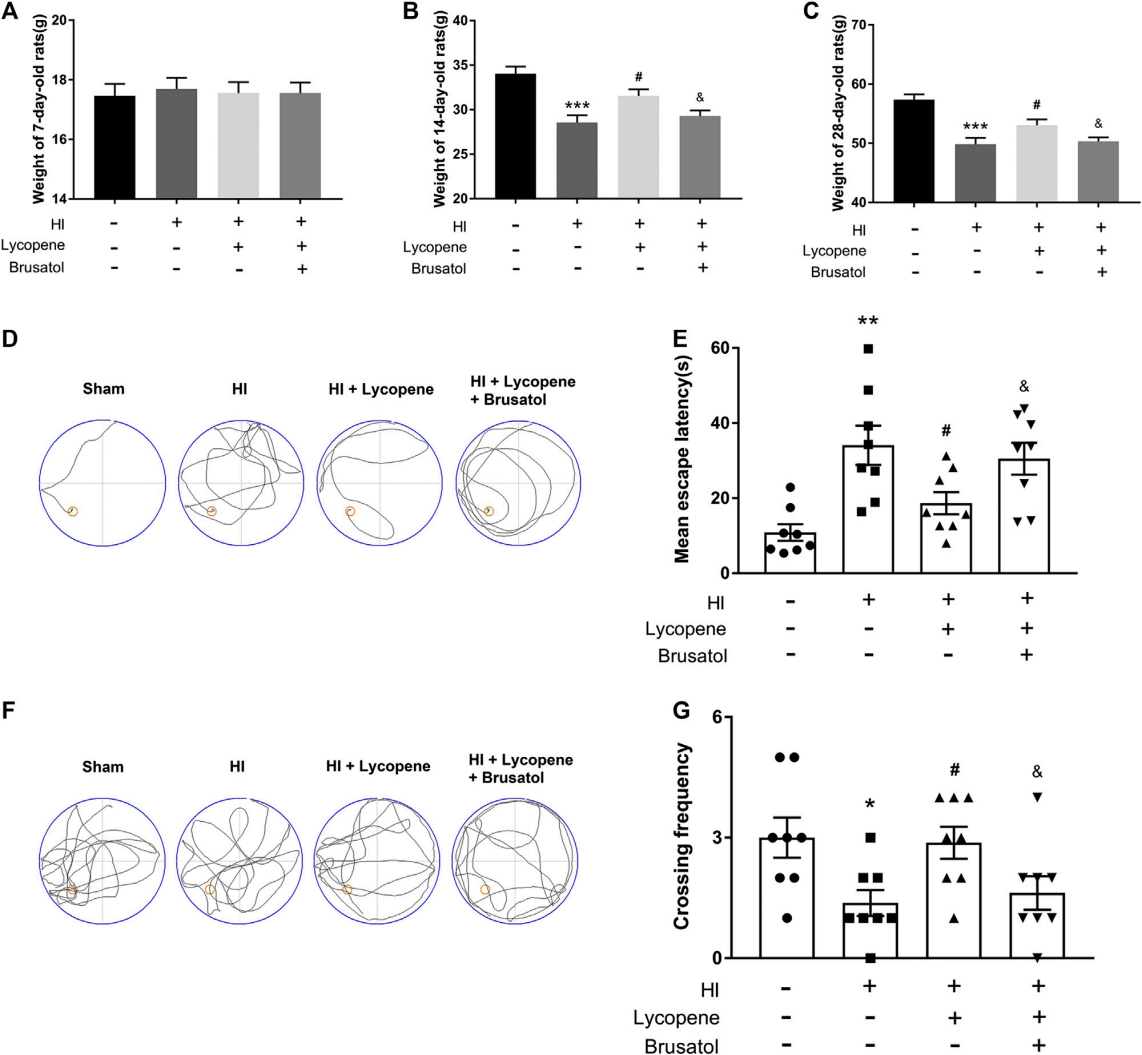
Treatment with Lycopene (Lyc) ameliorated the bodyweight and learning and memory ability of rats after HI injury. **(A)** The weights of rats from different groups are compared on day 7. All data are presented as mean ± SEM, n = 10. **(B)** The weights of rats from different groups are compared on day 14. ***p < 0.001 vs. the sham group; ^#^ *p* < 0.05 vs. the HI group; ^&^ *p* < 0.05 vs. the HI + Lyc group. All data are presented as mean ± SEM, n = 10. **(C)** The weights of rats from different groups are compared on day 28. ***p < 0.001 vs. the sham group; ^#^ *p* < 0.05 vs. the HI group; ^&^ *p* < 0.05 vs. the HI + Lyc group. All data are presented as mean ± SEM, n = 8. **(D)** Representative of swim route traces of rats from different groups. **(E)** Quantitative analysis of mean escape latency of Morris Water Maze tests in different groups of rats. **p < 0.01 vs. the sham group; ^#^ *p* < 0.05 vs. the HI group; ^&^ *p* < 0.05 vs. the HI + Lyc group. All data are presented as mean ± SEM, n = 8. **(F)** Representative images of swim route traces of rats from different groups after removal of the platform. **(G)** Quantitative analysis of the frequency of crossing the original platform location in the 60 s. *p < 0.05 vs. the sham group; ^#^ *p* < 0.05 vs. the HI group; ^&^ *p* < 0.05 vs. the HI + Lyc group. All data are presented as mean ± SEM, n = 8.

Additionally, we measured the bodyweight of the pups at 7, 14, and 28 days. The weights of all the four group were no significant differences before the HI injury. However, at day 14 and 28, the weights of the HI group were lower than the sham group. Furthermore, the weights of the Lyc treatment group were higher than the HI injury group. The HI + Lyc + Brusatol group inhibited the protective effect of Lyc in HI injury (Figures 5A–C). To demonstrate the therapeutic effect of Lyc on learning and memory function in rats after the HI brain injury, we conducted the Morris Water Maze test. Data from the spatial acquisition test suggested that the learning ability of the rats declined after HI brain injury, resulting in prolonged mean escape latency. However, rats in the HI + Lyc group spent less time looking for the hidden platform than the rats in the HI group (Figures 5D,E). As shown in Figures 5F,G, compared to the sham group, rats in the HI group had a lower crossing frequency. However, the Lyc treatment group were able to significantly reverse this trend. While the trend of the HI + Lyc + Brusatol group was similar to the HI group. These data indicated that Lyc ameliorates learning and memory functions in HI injury rats.

### Lycopene Attenuates Oxygen-Glucose Deprivation-Induced Apoptosis in Primary Cortical Neurons

**FIGURE 6:**
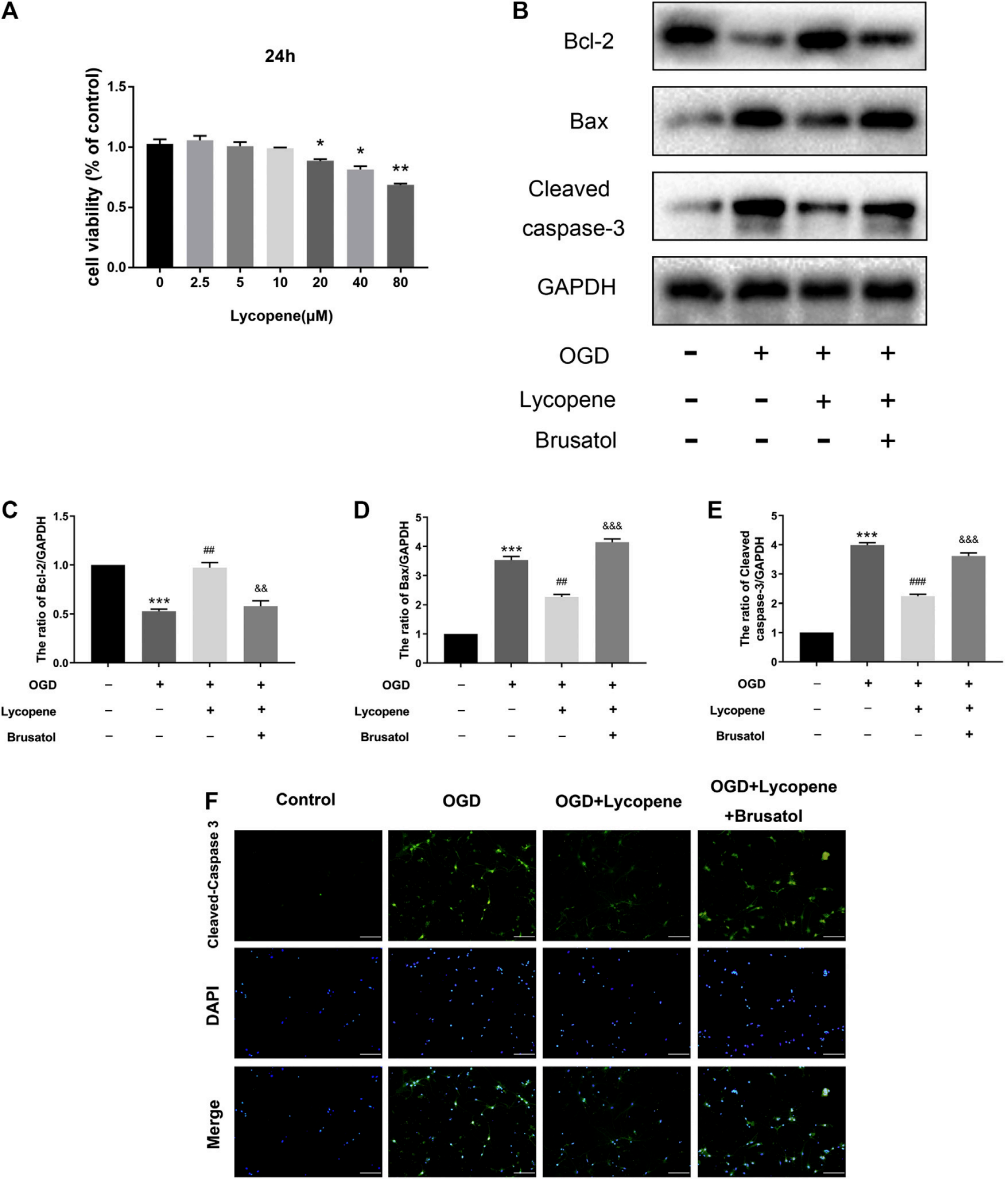
Treatment with Lycopene (Lyc) alleviated oxygen-glucose deprivation (OGD)-induced apoptosis in primary cortical neurons. **(A)** Using CCK8 analysis, the cytotoxic effect of Lyc (0, 2.5, 5, 10, 20, 40 and 80 μM) on primary cortical neurons. *p < 0.05, **p < 0.01 vs. the control group. All data are presented as mean ± SEM, n = 3. **(B)** The protein levels of Bcl-2, Bax and Cleaved caspase-3 were assessed by the Western blot. **(C,E)** Quantitative analysis of Bcl-2, Bax and Cleaved caspase-3. ***p < 0.001 vs. the control group; ^##^ *p* < 0.01 and ^###^ *p* < 0.001 vs. the OGD group; ^&&^ *p* < 0.01 and ^&&&^ *p* < 0.001 vs. the OGD + Lyc group. All data are presented as mean ± SEM, n = 3. **(F)** The representative fluorescence image of Cleaved caspase-3 with DAPI (nuclei). Scale bar = 100 μm.

Apoptosis is thought to be one of the most important pathological changes in the development and progression of HIE and OGD/R-induced neuronal injury. We used the CCK-8 assay to measure the Lyc cytotoxicity of primary cortical neurons. In brief, neurons were treated with various concentrations of Lyc (0, 2.5,5, 10, 20, 40, 80 µM) for 24 h. As shown in Figure 6A, no cytotoxic effect of Lyc was found at the doses of 0–10 µM. On the other hand, cell viability significantly decreased at 20 µM after 24 h. Therefore, 10 μM was utilized for the following experiments. Western blot results showed that the expression of anti-apoptotic proteins (Bcl-2) decreased, while that of pro-apoptotic proteins (Bax, Cleaved caspase-3) increased with OGD treatment. Treatment with Lyc was able to significantly reverse this trend. However, the anti-apoptotic effect of Lyc was inhibited by Brusatol (Figures 6B–E). Furthermore, we performed immunofluorescence staining to detect the anti-apoptotic effect of Lyc in OGD-induced apoptosis in primary cortical neurons. As shown in Figure 6F, the expression of cleaved caspase-3 in the OGD group was higher than the control group, whereas Lyc down-regulated the expression of cleaved caspase-3. However, Brusatol was able to abolish the protective effect of Lyc. Collectively, these data suggest that Lyc exerted an anti-apoptotic effect in OGD-induced primary cortical neurons.

## Discussion

HI is a severe condition of brain dysfunction that causes neonatal mortality and morbidity (Zalewska et al., 2015). Currently, there are no effective clinical therapies except for therapeutic hypothermia, which has been regarded as the only reliable standard therapy for infants with HIE (Silveira and Procianoy, 2015). Induction of neuroprotection is a crucial therapeutic strategy for the treatment of perinatal HIE (Johnston et al., 2011). However, the neuroprotective characteristics of these treatments are limited in their ability of promote tissue repair. Therefore, there is a need to find new synergistic therapies to improve neonatal HI brain injury.

Prior studies have shown that HIE is related to inflammation, apoptosis, and oxidative stress (Northington et al., 2011). Inflammatory cytokines are proven to play an important role in the progression of HIE (Barks et al., 2007). Johnson et al. found that inflammation can aggravate neurological outcomes in HIE (Johnson et al., 2016). Additionally, studies have reported that inflammatory cytokines were expressed in HI injury, including TNF-α, IL-1β, IL-6, etc (Šumanović-Glamuzina et al., 2017). Girard et al. found that administration of the anti-IL-1 receptor can improve the neuroprotective effects in HI brain injury. iNOS, one of the nitric oxide synthase (NOS) family of enzymes, which could promote the synthesis of NO (Girard et al., 2012). In current years, apoptosis has been demonstrated to have an important role in HI brain injury. Bcl-2, a member of the Bcl-2 family, protects neurons from apoptosis. To the best of our knowledge, caspase-3 is one of the most representative indicators of apoptosis, and cleaved caspase-3 is released at high levels, specifically following HI (Feng et al., 2014; Liu et al., 2018). A substantial number of studies have confirmed that apoptosis causes brain damage after hypoxia-ischemia, leading to massive cell loss and neurodegeneration (Rocha-Ferreira and Hristova, 2016). Thus, inducing a decrease in apoptosis has been considered a therapeutic strategy against HI injury in newborns. It has been found that oxidative stress is a contributor to ischemic brain injury (Warner et al., 2004). Mitochondria membrane potential and integrity were altered during the first hours after the HI and reactive oxygen species (ROS) activity is increased 12 h after the injury in the brainstem (Ferriero, 2001). Oxidative stress causing mitochondrial dysfunction and ROS activity were remarkably increased (Revuelta et al., 2015). After hypoxia-ischemia injury, generated free radicals which lead to oxidative stress and ultimate causes neuronal damage (Burchell et al., 2013). Low levels of antioxidants, along with a high metabolic rate and abundant lipid levels, makes brain cells highly sensitive to lipid peroxidation and oxidative damage (Chang et al., 2011).

In current years, some antioxidants have exerted a protective effect on hypoxia-ischemia (Llano et al., 2019). Nrf2 is a redox-sensitive transcription factor that is responsible for the antioxidant defense system by regulating the expressions of various oxidant stress-related enzymes, including superoxide dismutase (SOD) and HO-1 (Thimmulappa et al., 2006). Current evidence indicates that the Nrf2/NF-κB pathways can inhibit neuronal apoptosis after HI brain injury (Liu et al., 2018). Nrf2 is essential in energy metabolism and nerve cell development (Esteras et al., 2016). He et al. found that the Nrf2/NQO-1/HO-1/NF-κB signaling pathway was a critical in CI/R-induced oxidative stress, cell injury, and apoptosis (He et al., 2020). Moreover, Long Yang et al. uncovered that Astragaloside IV alleviates the brain damage induced by subarachnoid hemorrhage via PI3K/Akt/NF-κB signaling pathway (Yang et al., 2020).

Lyc, a kind of carotenoid, is a popular food ingredient drug at present. Its anti-inflammatory, anticancer, and antioxidative effects *in vivo* and *in vitro* have been reported in many articles (Olson and Krinsky, 1995; Condori et al., 2020). In addition, in the daily diet, a large part of Lyc comes from tomatoes, watermelons, pink grapefruit, and other red vegetables or fruits (Islamian and Mehrali, 2015). Meanwhile, compared to fresh tomatoes, it has been pointed out that food processing and cooking of tomatoes can improve the bioavailability of Lyc (Poor et al., 1993). Previous studies had shown that Lyc can inhibit the activation of the NF-κB signaling pathway by activating the Nrf2 pathway. Dong et al. found that Lyc attenuates LPS-induced liver injury by inactivating NF-κB/COX-2 and upregulating Nrf2/HO-1 activation (Dong et al., 2019). Dai et al. revealed that Lyc could ameliorate colistin-induced nephrotoxicity in mice via activation of the Nrf2/HO-1 pathway (Dai et al., 2015).

*In vivo* experiments of this study, we found that Lyc significantly decreased the brain infarct volume, attenuated apoptosis, inhibited inflammatory response, recovered histomorphology, increased body weights and improved behavior disorders after HI injury. Furthermore, Lyc exerts its neuroprotective effects after HI brain injury in neonatal rats via the Nrf2/NF-κB signaling pathway, and that the Lyc-mediated Nrf2/NF-κB signaling pathway can be inhibited by Brusatol (an Nrf2 inhibitor). Moreover, we investigated the protective of Lyc in OGD-induced cortical neurons that the results were concordant with Lyc *in vivo* result. We found that Lyc reduced the level of pro-apoptotic proteins (Bax, Cleaved caspase-3) and increase the level of anti-apoptotic proteins (Bcl-2).

In summary, theses data provide evidence that Lyc can attenuate HI brain injury in neurons or the nervous system by down-regulating apoptosis and inflammation through the Nrf2/NF-kB signaling pathway using both *in vivo* and *in vitro* experiments. Due to the complexity of the pathogenesis of HIE, accurate and effective therapies have not yet been developed, Lyc may be an inexpensive and effective alternative to other medicines.

## Conclusion

In conclusion, we demonstrated that Lyc can decrease the infarct volume, recovered tissue morphological, and improved learning and motor disabilities after the HI brain injury in neonatal rats. Through both *in vivo* and *in vitro* experiments, we demonstrated that Lyc can attenuate apoptosis and suppress the inflammatory response via the Nrf2/NF-kB signaling pathway. Taken together, our findings suggest that Lyc may be a potential therapeutic strategy for treatment of HI brain injury.

## Data Availability Statement

The raw data supporting the conclusions of this article will be made available by the authors, without undue reservation.

## Ethics Statement

The animal study was reviewed and approved by the Animal Care and Use Committee of Wenzhou Medical University.

## Author Contributions

CF, XF, PL and ZL designed the research study. YZ, BC, WL, and KL found and read relevant literatures. CF, JZ, SC and ZL designed the experiments. CF, YZ, JZ, BC and WL performed experiments. CF, YZ, JZ and KL analyzed data to form graphs. CF wrote the manuscript. XF, PL, and ZL helped to modified the manuscript.

## Funding

This study was funded by the National Natural Science Foundation of China (No. 81771624), the Beijing Medical and Health Foundation (No. B17137) and Wenzhou Municipal Science and Technology Bureau (No. Y20190001).

## Conflicts of Interest

The authors declare that the research was conducted in the absence of any commercial or financial relationships that could be construed as a potential conflict of interest.
